# Evaluation of a new chemical nematicide, fluazaindolizine (ReklemelTM active), for plant-parasitic nematode management in bermudagrass

**DOI:** 10.21307/jofnem-2021-043

**Published:** 2021-04-01

**Authors:** Will L. Groover, Kathy S. Lawrence

**Affiliations:** 559 Devall Dr. CASIC Building, Auburn, AL, Auburn University 36849

**Keywords:** *Belonolaimus*, *Meloidogyne*, Root-knot nematode, Sting nematode, Turfgrass

## Abstract

Plant-parasitic nematodes are a major pest of turfgrass in the United States, yet there are few options for successful management. Most current management strategies rely on the use of a limited number of chemical nematicides, so finding a new management option for nematode suppression would be extremely valuable for turfgrass managers. The aim of this study is to evaluate a new nematicide, fluazaindolizine (Reklemel™ active), for its ability to reduce plant-parasitic nematode population density and improve turfgrass quality. Separate research trials were conducted on bermudagrass infested with *Belonolaimus longicaudatus* and *Meloidogyne incognita* in greenhouse, microplot, and field settings over 2018 and 2019. Both greenhouse evaluations demonstrated multiple rates of fluazaindolizine reduced *B. longicaudatus* population density, and one of the two *M. incognita* trials showed multiple rates of fluazaindolizine reduced nematode population density. Fluazaindolizine was also effective at reducing population density of both *B. longicaudatus* and *M. incognita* in microplot settings for both 2018 and 2019, and a significant improvement in turf quality was observed for both visual turfgrass ratings and NDVI. Field trials demonstrated a significant reduction for both *B. longicaudatus* and *M. incognita* population density by multiple rates of fluazaindolizine, but no significant differences in turf quality ratings were observed. Overall, fluazaindolizine shows promise as a chemical nematicide for plant-parasitic nematode management on turfgrass.

Turfgrass is commonly grown throughout the United States for a wide range of uses, including golf courses, pastures, homeowner lawns, sod production, and institutional facilities (Breuniger et al., 2013). Economically, turfgrass has been estimated to have a total revenue of over $62 billion dollars, and geographically covers over 160,000 km^2^ of land (Haydu et al., 2005; Milesi et al., 2005). In the southeastern US, bermudagrass (*Cynodon* spp.) is the most commonly grown perennial warm-season turfgrass (Taliaferro, 1995). The warm and humid weather of the region makes this the ideal geography for bermudagrass growth and production.

One of the major pest issues for bermudagrass management in the southeast is plant-parasitic nematodes. A 2005 survey of golf courses in Florida found that over 80% of courses sampled were infested with plant-parasitic nematodes at potentially damaging levels (Crow, 2005). In Alabama, at least 10 genera of plant-parasitic nematodes have been recovered in routine assays from Alabama turfgrass, with many of these being found at damaging levels (Mullen, 1998; Sikora et al., 2001). Zeng et al. (2012) identified over 24 unique plant-parasitic nematode species on over 111 golf courses throughout North and South Carolina. Nematode damage occurs as they feed on the root system, leading to wilting, chlorosis, and thinning of the turf often in irregularly shaped patches (Crow and Han, 2005). This feeding inhibits root growth and development, leading to potential reductions in root biomass, water uptake, and nutrient absorption. With the high potential for significant damage to turf by plant-parasitic nematodes, timely management is extremely important, especially on highly maintained turfgrass. The primary strategy for nematode management is through a limited number of chemical nematicides.

Since its registration in 1973, fenamiphos (Nemacur, Bayer CropScience, St. Louis, MO) has dominated the turfgrass industry as the most frequently used nematicide (Keigwin, 2014). However, production of this product was halted in 2007 and it is currently not available for use. With this ban, turfgrass nematode management has shifted to newer and safer non-fumigant nematicides. One such example is abamectin (Divanem, Syngenta Crop Protection, Greensboro, NC). Abamectin has been shown to be effective against plant-parasitic nematodes on turf in the southeast (Blackburn et al., 1996; Crow, 2014). Fluensulfone (Nimitz Pro G, Adama, Pasadena, TX), a nematicide labeled for turfgrass in 2017, has also shown promise against plant-parasitic nematodes in turfgrass (Crow et al., 2017). A third commonly used chemical nematicide that was released for use on turfgrass starting in late 2016 is fluopyram (Indemnify, Bayer CropScience, Research Triangle Park, NC). Research trials conducted to evaluate fluopyram’s efficacy as a turfgrass nematicide have shown promise with a long residual of control (Baird et al., 2017; Crow et al., 2017). While each of these nematicides have been proven to provide benefit to plant-parasitic nematode management in turfgrass, recent research has also shown that relying too heavily on one nematicide has the potential to hurt soil health (Thoden et al., 2020; Waldo et al., 2019). Thus, finding new chemical nematicides to add to an integrated pest management program is always a valuable addition for turfgrass plant-parasitic nematode management.

One potentially useful chemical nematicide for turfgrass is fluazaindolizine (Reklemel™ active), a novel sulfonamide recently discovered to have nematicidal properties (Corteva Agriscience, Indianapolis, IN) (Lahm et al., 2017; Thoden and Wiles, 2019). Assessments of fluazaindolizine in vitro have shown its ability to significantly reduce motility and activity of *Meloidogyne incognita* juveniles compared to untreated juveniles, and greenhouse assays of fluazaindolizine on tomato have lowered *M. incognita’s* reproductive factor (Rf = final population density/initial population density) (Wram and Zasada, 2019). Fluazaindolizine has also been shown to be effective as a nematicide treatment over a multi-year field study on *M. incognita* infested carrots (Becker et al., 2019). Hajihassani et al. (2019) found that fluazaindolizine was effective in reducing the root gall index and *M. incognita* population density, as well as providing a consistent yield increase compared to an untreated control in cucumber trials. Fluazaindolizine has also demonstrated efficacy at reducing *M. incognita* root gall index, eggs per gram of root, and nematode reproductive factor in tomato compared to an untreated control (De Oliveira Silva et al., 2019).

With such a limited number of chemical nematicide options available for plant-parasitic nematode management on turfgrass, fluazaindolizine would be a beneficial addition for nematode management. Thus, the ability of fluazaindolizine to reduce both *M. incognita* and *Belonolaimus longicaudatus* population density on bermudagrass was evaluated. The overall objective of this study was to evaluate the potential of fluazaindolizine as a chemical nematicide for turfgrass in greenhouse, microplot, and field settings. The determination of fluazaindolizine efficacy as a turfgrass nematicide was evaluated by both its ability to reduce plant-parasitic nematode population density, and its ability to improve overall turf health.

## Materials and methods

### Nematicide treatments

The fluazaindolizine test substance used in these studies was a candidate suspension concentrate formulation containing 500 g/L of the active ingredient. Three rates of fluazaindolizine were evaluated for their ability to reduce both *M. incognita* and *B. longicaudatus* population density throughout the study. Fluopyram (Indemnify; Bayer CropScience, St. Louis, MO) was included as a chemical control, and an additional treatment of tap water was used as a negative control. Treatments for the experiments were (1) 4 applications of fluazaindolizine at a rate of 1.1 kg ai/ha at 0, 4, 8, and 12 weeks after trial initiation, (2) 2 applications of fluazaindolizine at a rate of 2.25 kg ai/ha (weeks 0 and 8), (3) 1 application of fluazaindolizine at a rate of 4.5 kg ai/ha (week 0), (4) 2 applications of fluopyram at 0.25 kg ai/ha (weeks 0 and 8), and (5) an untreated tap water control.

### Greenhouse evaluations

Two separate greenhouse trials were conducted in 2018 and repeated in 2019 at the Plant Science Research Center (PSRC) at Auburn University, Auburn, AL. One trial evaluated the efficacy of fluazaindolizine on *B. longicaudatus*, and the other *M. incognita*. In all, 500 cm^3^ polystyrene pots were filled with 100% sand. Each pot was then seeded with 1 gram of ‘Princess 77’ bermudagrass seed. Pots were watered daily as needed to allow for germination, and given 6 weeks for root establishment before trial initiation. Pots were also fertilized every 14 days using 24-8-16 (N-P_2_O_5_-K_2_O) at a rate of 0.5 kg N per 100 m^2^ per growing month and trimmed once a week to a height of 2.54 cm. Lighting was supplied via 1,000-watt halide bulbs producing 110,000 lumens for 14 h per day and temperatures in the greenhouse ranged from 24 to 35°C. Nematicide treatments were applied as foliar sprays via a handheld spray bottle, and treatments were diluted so that two sprays from the bottle was the calibrated rate.

### Greenhouse nematode inoculum

*Meloidogyne incognita* race 3, originally isolated from an infested field at the Plant Breeding Unit (PBU) at E.V. Smith Research Center of Auburn University and maintained on corn “Mycogen 2H723” (Corteva AgriScience, Indianapolis, IN) in 500 cm^3^ polystyrene pots in the greenhouse, was used as inoculum in the first experiment (Groover et al., 2019). To obtain the nematode population, the eggs were extracted from the corn roots following a modified version of the methodology of Hussey and Barker (1973). The root mass was placed in a 0.625% NaOCl solution and shaken for 4 min at 1 g-force on a Barnstead Lab Line Max Q 5000 E Class shaker (Conquer Scientific, San Diego, CA). Roots were scrubbed by hand, and the eggs were collected on a 25-µm pore sieve and washed into a 50 mL centrifuge tube. The contents were centrifuged at 427 g-forces for 1 min in a 1.14 specific gravity sucrose solution based on Jenkins (1964) methodology. Eggs, now located in the supernatant of the sucrose solution, were recollected on a 25-µm pore sieve, rinsed with water to remove sucrose from eggs, and their presence confirmed via a Nikon TSX 100 inverted microscope at 40-x magnification. The eggs were placed in a modified Baermann funnel (Castillo et al., 2013) on a slide warmer (Model 77; Marshall Scientific, Brentwood, NH) and incubated at 31°C for 5 to 7 days to obtain second-stage juveniles (J2). The J2 were collected on a 25-µm pore sieve, transferred to 1.5 mL micro-centrifuge tubes, centrifuged at 5,000 g for 1 min, rinsed with sterile distilled water, and centrifuged again at 5,000 g for 1 min. The J2 solution was adjusted to 1,000 J2 per 1 mL of water, and 2 mL of solution containing 2,000 J2 were pipetted into each pot.

For the other greenhouse experiment, *B. longicaudatus*, maintained on ‘Princess 77’ bermudagrass in 500 cm^3^ polystyrene pots, was used as inoculum. To obtain the nematode population, total soil from each pot was collected on a 25-µm pore sieve and nematodes were extracted using the modified sucrose centrifugal flotation technique as described above. The final *B. longicaudatus* population was collected on a 25-µm pore sieve and transferred to a 1.5 mL micro-centrifuge tube, centrifuged at 5,000 g for 1 min, rinsed with sterile distilled water, and centrifuged again at 5,000 g for 1 min. The nematode suspension was then adjusted to 20 nematodes per 1 mL of water, and 2 mL of solution containing 40 *B. longicaudatus* were pipetted into each pot.

### Greenhouse data collection

All experiments were arranged in a randomized complete block design (RCBD) with five replications. Turfgrass vigor was calculated using the National Turfgrass Evaluation Program (NTEP) guidelines (Parsons et al., 2015). Visual ratings consisted of a 1–9 rating scale, where 1 was very poor quality turf, 6 was minimal acceptable turf quality, and 9 was exceptional turf quality (Morris and Shearman, 2014). The normalized difference vegetation index (NDVI) measurements using a Greenseeker Handheld Crop Sensor (Trimble Inc., Sunnyvale, CA) were taken on a weekly basis for each trial in both years. Experiments were harvested 84 days after the first nematicide applications were made. Nematode samples from each pot were collected at the completion of the trial. Soil collected from each pot was collected on a 25-µm pore sieve was then used to calculate final nematode populations for both *M. incognita* and *B. longicaudatus* trials using the modified centrifugal flotation technique as previously described (Jenkins, 1964). Extracted nematodes were enumerated at 40-x magnification using an inverted TS100 Nikon microscope and quantified as total nematodes per pot and as nematodes per gram of root fresh weight (RFW).

### Microplot evaluations

The same treatments used in the greenhouse experiments were used for trials conducted in a microplot setting. Microplot trials were conducted in two separate years (2018 and 2019) at the PSRC in Auburn, AL. All plots were arranged in a RCBD with five replications. For these trials, 26.5-liter plastic tree pots were used as microplots. Pots were nested one on top of the other with a brick in between to limit root growth by air pruning. The nested pot design was buried in the ground with one inch of the pot above the soil surface. Microplots were then filled with 100% medium-coarse sand (0.25–1.0 mm). ‘Tifway’ hybrid bermudagrass sod was established in each plot and given 10 weeks for root establishment. At the end of the 10-week period, *M. incognita* eggs were inoculated at a rate of 50,000 eggs per pot on a weekly basis for 4 weeks to build up nematode population density in half of the microplots. The remaining microplots received an inoculation rate of 100 *B. longicaudatus* nematodes per pot on a weekly basis for 4 weeks. After the 4-week inoculation period, a 100-cm^3^ soil sample was taken from each plot to confirm *M. incognita* or *B. longicaudatus* presence. Treatments were applied as foliar sprays via a handheld spray bottle, and treatments were diluted so that two sprays from the bottle was the calibrated rate for each treatment per microplot. Treatment regimen was identical in application rates and timing as greenhouse experiments. Each microplot received water at 30 mL/min by an automated drip irrigation system adjusted throughout the season to run for 30 min twice a day every other day. Grass was trimmed twice a week to a height of 2.5 cm.

### Microplot data collection

Visual turfgrass ratings and NDVI ratings with the handheld sensor were taken throughout the trial starting at trial initiation followed by 7-day increments. Nematode population density was determined at three time points during the trial in 2018 (June 29, August 24, and September 28), and four time points in 2019 (July 22, August 19, September 23, and October 21). Nematode extraction and population density determination was performed as previously described.

### Field evaluations

In 2018, a field trial evaluating fluazaindolizine as a turfgrass nematicide for *Belonolaimus longicaudatus* was conducted on common bermudagrass grown under fairway conditions at the Auburn University Gulf Coast Research and Extension Center (GCREC) in Fairhope, AL after confirming *B. longicaudatus* presence. The treatments for both greenhouse and microplot trials were also used for the field evaluation. Individual plots were 1.5 meters by 3 meters, with a 0.6-meter border between adjacent plots. The trial was set up as a randomized complete block design with five replications. Each treatment was mixed with water to a total volume of one gallon and sprayed on the plots with a CO_2_-powered backpack sprayer (R&D Sprayers, Bellspray, Inc., Opelousas, LA).

Two field trials were conducted in 2019 for evaluating fluazaindolizine as a turfgrass nematicide. Trials were conducted at GCREC in the same location as 2018 for *Belonolaimus longicaudatus* evaluations, and an adjacent location with a confirmed population density of *Meloidogyne* spp. The treatment list remained the same as previous studies for both nematode trials.

### Field data collection

Data collection was the same for both years. Visual turfgrass ratings and NDVI values with a handheld sensor were taken at the start of the experiment, followed by approximately 2-week intervals throughout the trial as previously described. Along with these measurements, field trials were mapped with a DJI Phantom 4 Professional drone (SZ DJI Technology Co., Nanshan District, Shenzhen, China). This drone was equipped with a MicaSense RedEdge-M sensor (MicaSense, Inc., Seattle, WA). Image processing was performed with Pix4Dmapper (Pix4D, Prilly, Switzerland), and image analysis was carried out with ArcMap (Esri, Redlands, CA), providing NDVI and NDRE ratings for each plot. Nematode samples were taken at three time points in 2018 (August 7, September 4, and October 2) and four time points in 2019 (July 25, August 22, September 24, and October 23), approximately at 4-week intervals. Nematode samples consisted of seven 2.22-cm-diameter × 10-cm-deep cores from each plot. Samples were thoroughly mixed, and nematodes were extracted from a 100 cm^3^ subsample and enumerated as previously described.

### Statistical analysis

Data collected from greenhouse, microplot, and field evaluations were analyzed using the PROC GLIMMIX procedure (SAS version 9.4, SAS Institute, Cary, NC). Dependent variables included *B. longicaudatus* per 100 cm^3^ soil, *M. incognita* per 100 cm^3^ soil, visual turf quality, and NDVI. The fixed effect was nematicide treatment, and random effects included replication, test repeat, and location. Student panels were generated to determine the normality of residuals. For greenhouse and microplot evaluations, LS-means were compared between treatments by Tukey’s multiple range test for each evaluation date (*P ≤ *0.05). For the microplot evaluation, visual turfgrass quality and NDVI ratings were compared statistically by linear correlation at each nematode sample date (*P ≤ *0.05, *P ≤ *0.01, *P ≤ *0.001).

## Results

### Greenhouse evaluations

**Figure 1:**
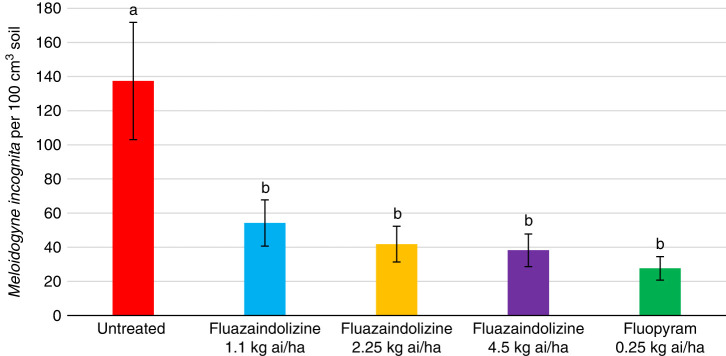
2018 *Meloidogyne incognita* population density in greenhouse evaluations 84 days after treatment (DAT). Rates were applied as follows: Fluazaindolizine 1.1 kg ai/ha at weeks 0, 4, 8, 12; 2.25 kg ai/ha at weeks 0, 8; 4.5 kg ai/ha at week 0; fluopyram 0.25 kg ai/ha at weeks 0, 8. Means of bars with the same letter above them are not significantly different (Tukey–Kramer, *P ≤ *0.05).

**Figure 2:**
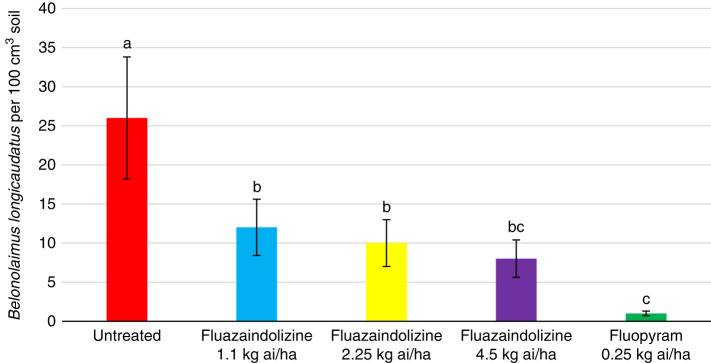
2018 *Belonolaimus longicaudatus* population density in greenhouse evaluations 84 days after treatment (DAT). Rates were applied as follows: Fluazaindolizine 1.1 kg ai/ha at weeks 0, 4, 8, 12; 2.25 kg ai/ha at weeks 0, 8; 4.5 kg ai/ha at week 0; fluopyram 0.25 kg ai/ha at weeks 0, 8. Means of bars with the same letter above them are not significantly different (Tukey–Kramer, *P ≤ *0.05).

**Figure 3:**
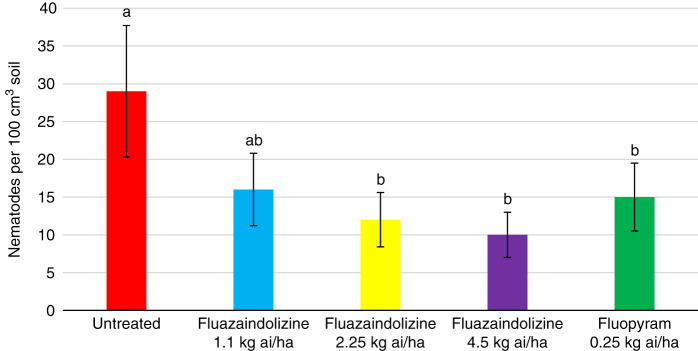
2019 *Belonolaimus longicaudatus* population density in Auburn, AL greenhouse evaluations 84 days after treatment (DAT). Nematicide rates were applied as follows: Fluazaindolizine 1.1 kg ai/ha at weeks 0, 4, 8, 12; 2.25 kg ai/ha at weeks 0, 8; 4.5 kg ai/ha at week 0; fluopyram 0.25 kg ai/ha at weeks 0, 8. Means of bars with the same letter above them are not significantly different (Tukey–Kramer, *P ≤ *0.05).

All nematicide treatments significantly reduced *M. incognita* population density compared to the untreated control in 2018 greenhouse evaluations (*P ≤ *0.05) (Fig. 1). However, despite seeing numerical reductions by all nematicide treatments in 2019, there were no significant differences between treatments (data not shown). In the *B. longicaudatus* greenhouse evaluations, all treatments significantly reduced nematode population density compared to the untreated control in 2018 (*P ≤ *0.05) (Fig. 2). Fluopyram also significantly lowered nematode population density compared to the 1.1 kg ai/ha and 2.25 kg ai/ha rates of fluazaindolizine, but was statistically similar to the 4.5 kg ai/ha rate of fluazaindolizine (*P ≤ *0.05). In 2019, the 2.25 kg ai/ha and 4.5 kg ai/ha rates of fluazaindolizine as well as the fluopyram control significantly reduced *B. longicaudatus* population density compared to the untreated control (*P ≤ *0.05) (Fig. 3).

### Microplot evaluations

**Figure 4:**
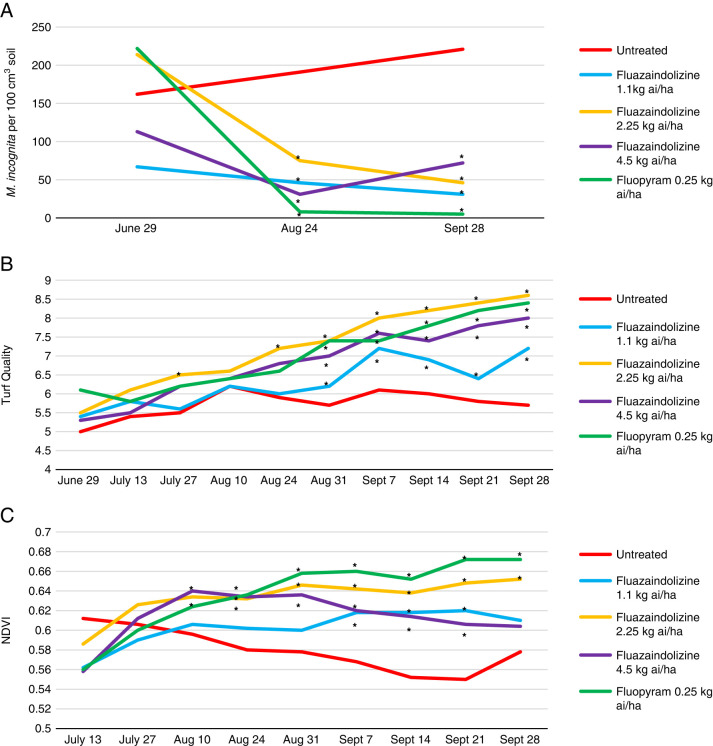
*Meloidogyne incognita* population density (A), visual turfgrass quality (B), and NDVI values (C) as affected by nematicide applications through 2018 bermudagrass microplot evaluations in Auburn, AL, 2018. Nematicide rates were applied as follows: Fluazaindolizine 1.1 kg ai/ha at weeks 0, 4, 8, 12; 2.25 kg ai/ha at weeks 0, 8; 4.5 kg ai/ha at week 0; fluopyram 0.25 kg ai/ha at weeks 0, 8. *Different from the untreated according to the pairwise comparison of each treatment to the untreated control (Tukey–Kramer; *P ≤ *0.05).

In the 2018 *M. incognita* microplot evaluations, all nematicide treatments significantly reduced population density compared to the untreated control at both the August and September evaluation date (*P ≤ *0.05) (Fig. 4A). For visual turfgrass quality, 7 of 10 evaluation dates saw significant improvement by at least one nematicide treatment compared to the untreated control, with the 2.25 kg ai/ha rate of fluazaindolizine having the numerically highest visual turfgrass rating at 8 of 10 evaluation dates (*P ≤ *0.05) (Fig. 4B). Six of the nine NDVI evaluation dates also saw a significant improvement by at least one nematicide treatment compared to the untreated control, with fluopyram having the numerically largest NDVI value at five of the nine evaluation dates (*P ≤ *0.05) (Fig. 4C).

**Figure 5:**
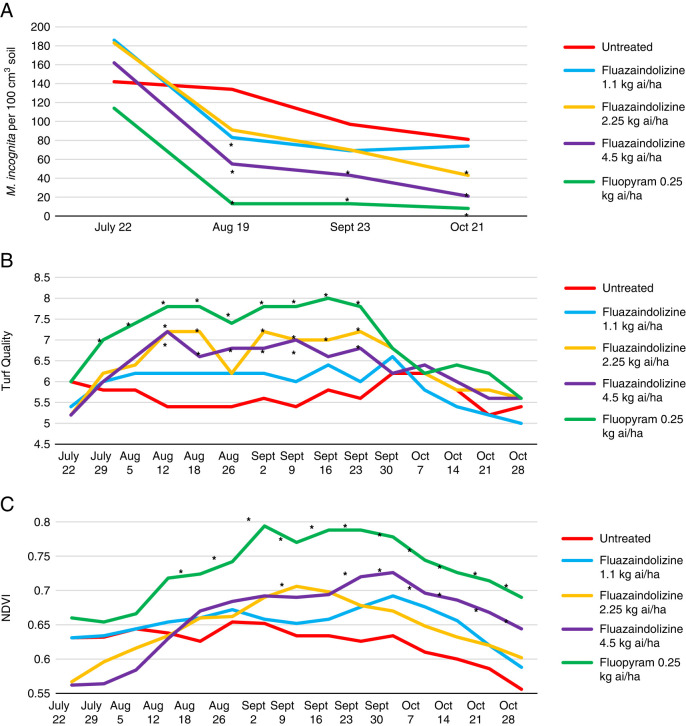
*Meloidogyne incognita* population density (A), visual turfgrass quality (B), and NDVI values (C) as affected by nematicide treatments in 2019 bermudagrass microplot evaluations, Auburn, AL. Nematicide rates were applied as follows: Fluazaindolizine 1.1 kg ai/ha at weeks 0, 4, 8, 12; 2.25 kg ai/ha at weeks 0, 8; 4.5 kg ai/ha at week 0; fluopyram 0.25 kg ai/ha at weeks 0, 8. *Different from the untreated according to the pairwise comparison of each treatment to the untreated control (Tukey–Kramer; *P ≤ *0.05).

In 2019, multiple nematicide treatments significantly impacted *M. incognita* population density in the microplot trial. The 4.5 kg ai/ha rate of fluazaindolizine and the fluopyram treatment significantly reduced *M. incognita* population density compared to the untreated control at the August, September, and October sample dates, and the 2.25 kg ai/ha rate of fluazaindolizine significantly lowered population density at the October sample date (*P ≤ *0.05) (Fig. 5A). Of the 15 turfgrass visual quality evaluation dates, significant differences between treatments were observed at 9 dates, with the fluopyram treatment consistently having the highest numerical visual turfgrass quality (*P ≤ *0.05) (Fig. 5B). This was closely followed by the 2.25 and 4.5 kg ai/ha rates of fluazaindolizine that had significantly improved visual turfgrass quality at 8 evaluation dates compared to the untreated control (*P ≤ *0.05). For the NDVI evaluations, significant differences between treatments were observed at all evaluation dates, with fluopyram having the numerically largest value throughout the entire trial (*P ≤ *0.05) (Fig. 5C).

**Figure 6:**
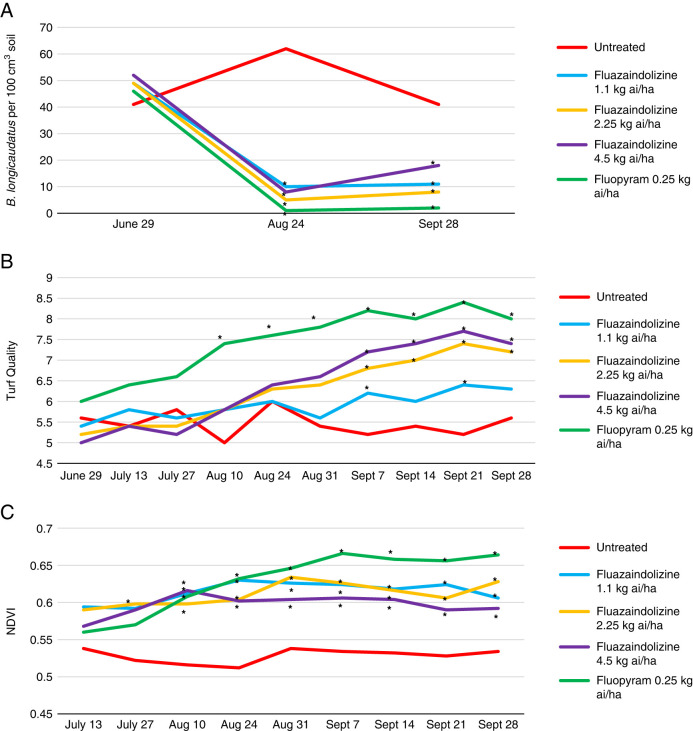
*Belonolaimus longicaudatus* population density (A), visual turfgrass quality (B), and NDVI values (C) as affected by nematicide treatments in 2018 bermudagrass microplot evaluations, Auburn, AL. Nematicide rates were applied as follows: Fluazaindolizine 1.1 kg ai/ha at weeks 0, 4, 8, 12; 2.25 kg ai/ha at weeks 0, 8; 4.5 kg ai/ha at week 0; fluopyram 0.25 kg ai/ha at weeks 0, 8. *Different from the untreated according to the pairwise comparison of each treatment to the untreated control (Tukey–Kramer; *P ≤ *0.05).

**Figure 7:**
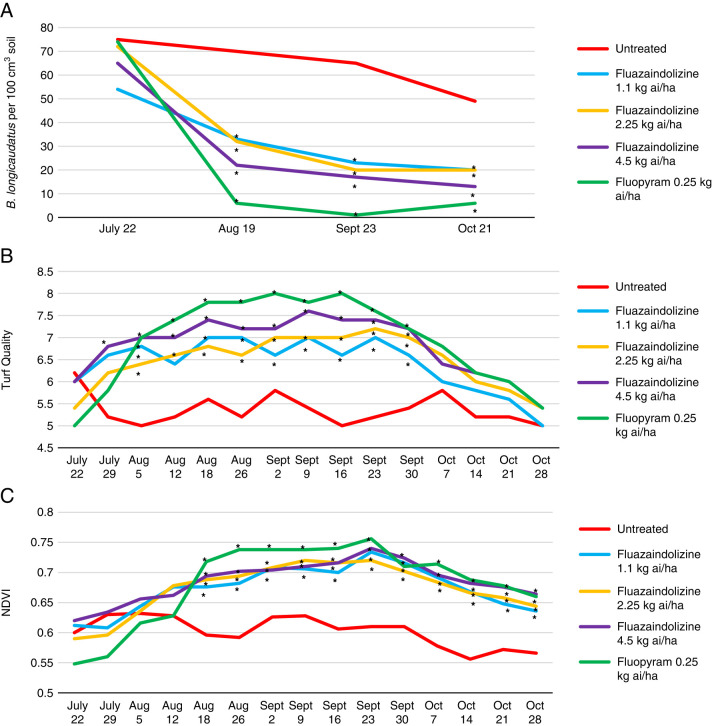
*Belonolaimus longicaudatus* population density (A), visual turfgrass quality (B), and NDVI values (C) as affected by nematicide treatments in 2019 bermudagrass microplot evaluations, Auburn, AL. Nematicide rates were applied as follows: Fluazaindolizine 1.1 kg ai/ha at weeks 0, 4, 8, 12; 2.25 kg ai/ha at weeks 0, 8; 4.5 kg ai/ha at week 0; fluopyram 0.25 kg ai/ha at weeks 0, 8. *Different from the untreated according to the pairwise comparison of each treatment to the untreated control (Tukey–Kramer; *P ≤ *0.05).

*Belonolaimus longicaudatus* population density was significantly reduced by all nematicides in the 2018 microplot trial at both evaluation dates after trial initiation (*P ≤ *0.05) (Fig. 6A). Visual turfgrass quality was also significantly improved by at least one nematicide treatment at 9 of 10 evaluation dates, with the largest improvement in visual quality in the later sample dates by fluopyram, the 2.25 kg ai/ha rate of fluazaindolizine, and the 4.5 kg ai/ha rate of fluazaindolizine (*P ≤ *0.05) (Fig. 6B). NDVI was significantly improved by all nematicide treatments at all except one evaluation date in the 2018 *B. longicaudatus* microplot trial (*P ≤ *0.05) (Fig. 6C). In the 2019 *B. longicaudatus* microplot trial, nematode population density was significantly reduced by all nematicides at the August, September, and October sample dates (*P ≤ *0.05) (Fig. 7A). This led to a significant increase in visual turf quality by all treatments compared to the untreated control at 10 of 15 evaluation dates, and a significant increase in NDVI by all treatments at 11 of 15 evaluation dates (*P ≤ *0.05) (Fig. 6B,C).

Significant linear correlations were observed in the microplot trials throughout 2018 and 2019 for both *M. incognita* and *B. longicaudatus* population density when compared to both visual turfgrass quality and NDVI (Table 1). In general, as nematode population density declined, both visual turfgrass quality and NDVI values increased. For *M. incognita* evaluations, one of the three nematode sample dates was significantly correlated with visual quality (*P ≤ *0.001) and NDVI (*P ≤ *0.01) in 2018, and all four nematode sample dates in 2019 (*P ≤ *0.05). For *B. longicaudatus* evaluations, two of the three sample dates had a significant correlation with both visual quality and NDVI in 2018 (*P ≤ *0.01), and all four nematode sample dates in 2019 (*P ≤ *0.001).

**Table 1.** Pearson correlation coefficients^†^ resulting from linear correlation of *Meloidogyne incognita* and *Belonolaimus longicaudatus* population density in microplot bermudagrass with visual turfgrass quality and NDVI values in Auburn, AL for 2018 and 2019.

	2018			
	June 29	August 24	September 28	
	*Meloidogyne incognita* population density			
Visual quality^‡^	0.05	0.05	−0.77***	
NDVI^§^	N/A	−0.24	−0.52**	
	*Belonolaimus longicaudatus* population density			
Visual quality	−0.08	−0.64***	−0.60**	
NDVI	N/A	−0.62***	−0.54**	
	2019			
	July 22	August 19	September 23	October 21
	*Meloidogyne incognita* population density			
Visual quality	−0.78***	−0.60**	−0.38*	−0.38*
NDVI	−0.77***	−0.49*	−0.51**	−0.47*
	*Belonolaimus longicaudatus* population density			
Visual quality	−0.73***	−0.78***	−0.75***	−0.75***
NDVI	−0.63***	−0.79***	−0.81***	−0.73***

Notes: †*,**,***Tests of linear correlation between variables were not significant or were significant at *P ≤ *0.05, *P ≤ *0.01, or *P ≤ *0.001, respectively. ‡Visual quality ratings were assigned on a 1 to 9 scale, where 1 = poorest turf quality, 6 = minimally acceptable turf quality, and 9 = exceptional turf quality. §NDVI = (840 nm reflectance – 668 nm reflectance) ÷ (840 nm reflectance – 668 nm reflectance) collected using a MicaSense RedEdge-M (MicaSense, Inc., Seattle, WA) sensor.

### Field evaluations

**Figure 8:**
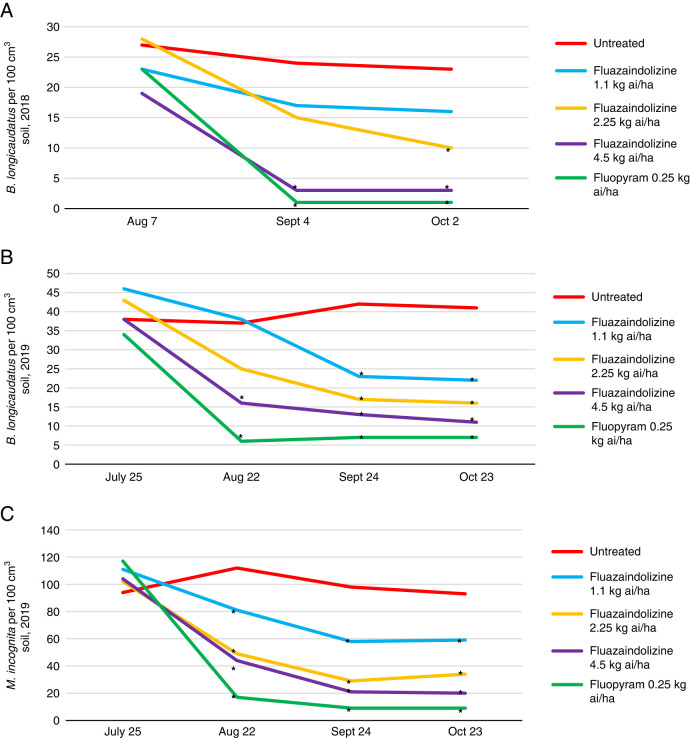
*Belonolaimus longicaudatus* population density in 2018 (A) and 2019 (B), and *Meloidogyne incognita* population density in 2019 (C) as affected by nematicide treatments in nematicide trials at Fairhope, AL. Nematicide rates were applied as follows: Fluazaindolizine 1.1 kg ai/ha at weeks 0, 4, 8, 12; 2.25 kg ai/ha at weeks 0, 8; 4.5 kg ai/ha at week 0; fluopyram 0.25 kg ai/ha at weeks 0, 8. *Different from the untreated according to the pairwise comparison of each treatment to the untreated control (Tukey–Kramer; *P ≤ *0.05).

For both 2018 and 2019 field evaluations, nematicide treatments had a significant impact on plant-parasitic nematode population density (Fig. 8). However, no visual symptoms were ever observed in the trial, thus no significant differences for visual ratings, handheld NDVI, or drone NDVI and NDRE values were reported over both years (data not shown). In 2018, the 4.5 kg ai/ha rate of fluazaindolizine and fluopyram both significantly reduced *B. longicaudatus* population density at the September and October sample dates, and the 2.25 kg ai/ha rate of fluazaindolizine significantly reduced population density at the October sample date (*P ≤ *0.05). In both the *B. longicaudatus* and *M. incognita* 2019 field trials, three of the four nematicide treatments significantly reduced nematode population density compared to the untreated control at the August evaluation date, and all nematicides significantly reduced population density at the September and October evaluation dates (*P ≤ *0.05) (Fig. 8B,C).

## Discussion

The results from both year’s greenhouse, microplot, and field evaluations of fluazaindolizine indicate that there is a strong potential for its use in plant-parasitic nematode management in turfgrass. Plant-parasitic nematode population density was significantly lowered when compared to an untreated control in all three settings. These significant population declines did overall reduce numbers of *B. longicaudatus* and *M. incognita* below the standard 10 and 80 per 100 cm^3^ of soil. In the microplot trials, both visual turfgrass quality ratings and NDVI were significantly improved by multiple rates of fluazaindolizine. Thus, with a reduction in both *B. longicaudatus* and *M. incognita* population density, and a general improvement in plant health, both efficacy criteria for fluazaindolizine evaluation were met.

Greenhouse evaluations showed strong nematicidal activity for fluazaindolizine in both *B. longicaudatus* and *M. incognita* trials. A numerical rate response was also observed, with higher rates of fluazaindolizine leading to lower final population density in all trials. While a significant reduction in *M. incognita* population density was not observed compared to the untreated control in the 2019 trial, no final population density for any of the evaluated fluazaindolizine rates were at levels that traditionally are of concern for a turfgrass manager in the southeastern United States (Sikora et al., 2001).

The results of the microplot trials also showed nematicidal activity against both *M. incognita* and *B. longicaudatus.* Unlike the greenhouse trials, a rate response was not consistently observed. In fact, in the 2018 *M. incognita* trial, while all treatments lowered population density below high risk levels, the monthly application of the 1.1 kg ai/ha rate of fluazaindolizine led to the lowest population density at the end of the trial of the three fluazaindolizine rates evaluated. This was not the case in 2019, as the 4.5 kg ai/ha rate of fluazaindolizine applied at the start of the trial resulted in the largest reduction in *M. incognita* population density. In the 2018 *B. lonigcaudatus* trial, two applications of the 2.25 kg ai/ha rate led to the lowest population density, with the 4.5 kg ai/ha rate treatment still having nematodes at above threshold levels. However, the 2019 *B. longicaudatus* trial showed a dose response trend, where the larger the initial application the larger the numerical reduction in nematode population density. While turf quality and NDVI were significantly improved by fluazaindolizine applications compared to the untreated control, there were rarely any evaluation dates where differences between the three rates were observed. Over all the microplot data, two applications of 2.25 kg ai/ha of fluazaindolizine and one application of 4.5 kg ai/ha of fluazaindolizine led to the most consistent improvement of turfgrass vigor compared to the untreated control, showing that these higher initial rates of the product may be the best option for plant-parasitic nematode management on turfgrass.

Field trials showed strong reductions of both *B. longicaudatus* and *M. incognita* population density by fluazaindolizine. While all nematicide rates and applications led to nematode population density reduction, the medium and high rates of fluazaindolizine as well as fluopyram most consistently lowered population density across all trials. However, despite confirming strong population density for both *M. incognita* and *B. longicaudatus* at the field sites for these trials, no visual symptoms were ever observed. Thus, while the higher rates of fluazaindolizine again had strong impacts on nematode density, no conclusions can be made on the effect of fluazaindolizine on plant vigor in the field setting.

This research confirms previous studies that have shown a similar efficacy of fluazaindolizine to *M. incognita* as a nematicide for reducing population density on crops including cucumber, carrot, and tomato (Becker et al., 2019; De Oliveira Silva et al., 2019; Hajihassani et al., 2019). However, while *M. incognita* is pathogenic to bermudagrass, it is not traditionally the main *Meloidogyne* species identified on turfgrass in the southeast (Crow, 2019; Ye et al., 2015; Zeng et al., 2012). For this research, *M. incognita* was used for trials because all research plots were artificially inoculated to obtain infested turfgrass, and *M. incognita* was available at high enough population density for successful inoculation. Moving forward, field locations with species more commonly found on turfgrass need to also be evaluated (*M. graminis* and *M. marylandi*) to confirm the findings of this study.

To our knowledge, this is the first published study on fluazaindolizine efficacy as a turfgrass nematicide. These findings indicate that while each rate evaluated was successful at lowering both *M. incognita* and *B. longicaudatus* population density, higher initial application rates of fluazaindolizine were more consistent at both lowering nematode population density and improving plant health quality. While these results show promise for this product, more studies need to be conducted. Overall, fluazaindolizine appears to have strong potential for including in a turfgrass nematode integrated management program.
